# Overcoming Trastuzumab–Pertuzumab Resistance and Optimizing Sequential Anti-HER2 Therapy in HER2-Positive Metastatic Breast Cancer

**DOI:** 10.3390/cancers18060932

**Published:** 2026-03-13

**Authors:** Yutaka Yamamoto

**Affiliations:** Department of Breast and Endocrine Surgery, Kumamoto University Hospital, 1-1-1 Honjo, Chuo-ku, Kumamoto 860-8556, Japan; yyamamoto@kumamoto-u.ac.jp; Tel.: +81-96-373-5521

**Keywords:** trastuzumab, pertuzumab, resistance, metastatic breast cancer

## Abstract

HER2-positive breast cancer accounts for 15–20% of all breast cancers. The introduction of anti-HER2 therapies has markedly improved the clinical outcomes; however, overcoming drug resistance in metastatic disease remains a major challenge. This review summarizes the multilayered mechanisms of resistance to trastuzumab and pertuzumab and outlines the rationale for sequential treatment strategies based on the emerging evidence. Resistance arises through diverse and often coexisting mechanisms, including structural alterations in the HER2 receptor (e.g., p95HER2 and HER2 mutations), constitutive activation of the PI3K–AKT–mTOR pathway, and engagement of bypass signaling through receptors such as HER3 and IGF-1R, as well as immune evasion and metabolic reprogramming. Given this complexity, the strategic sequencing of agents with distinct mechanisms of action is critical beyond first-line therapy. Trastuzumab deruxtecan demonstrates substantial antitumor activity through potent cytotoxic effects and a bystander effect, supporting its efficacy in tumors with intratumoral heterogeneity or downstream pathway activation. In contrast, tucatinib-based regimens represent an important option for patients with brain metastases and tumors expressing p95HER2. The ongoing development of novel antibody–drug conjugates and bispecific antibodies is expected to further advance personalized sequential therapy targeting composite resistance mechanisms.

## Simple Summary

Although anti-HER2 therapies have markedly improved outcomes in HER2-positive breast cancer, resistance to anti-HER2 therapy remains a major challenge in the metastatic setting. This review delineates the landscape of resistance to trastuzumab and pertuzumab—including p95HER2 expression, HER2 mutations, activation of the PI3K/AKT/mTOR pathway, bypass signaling, immune evasion, and metabolic reprogramming—and highlights the clinical necessity of rational sequential treatment strategies. In the second-line setting and beyond, careful sequencing of agents with distinct mechanisms of action is essential. Trastuzumab deruxtecan (T-DXd) demonstrates robust efficacy even in the context of intratumoral heterogeneity, whereas tucatinib-based regimens play a pivotal role in the management of brain metastases. Future advances in personalized medicine, driven by novel antibody–drug conjugates and bispecific antibodies, are expected to further overcome these resistance mechanisms.

## 1. Introduction

HER2-positive breast cancer accounts for approximately 15–20% of all breast cancer cases [1]. At the molecular level, it is characterized by the high expression of HER2-related genes, including *ERBB2* and *GRB7*, as well as genes associated with cellular proliferation [2]. Genome-wide analyses have demonstrated a high frequency of somatic alterations, including *TP53* mutations (75%) and *PIK3CA* mutations (43%), reflecting substantial genomic instability [3]. Without systemic therapy incorporating anti-HER2 agents in the postoperative setting, the clinical outcomes are poor [4].

Systemic therapy remains the cornerstone for both recurrent and de novo metastatic disease; however, optimizing the initial and subsequent treatment sequences is crucial for long-term survival [5]. The introduction of anti-HER2 monoclonal antibodies, including trastuzumab and pertuzumab, has markedly improved the treatment outcomes, significantly prolonging the progression-free and overall survival in both early-stage and metastatic disease [4]. Nevertheless, most patients with HER2-positive metastatic breast cancer (MBC) eventually develop resistance to these therapies, resulting in disease progression and limited subsequent treatment options [4,6,7].

In the DESTINY-Breast09 trial [8], the combination of trastuzumab deruxtecan (T-DXd) and pertuzumab demonstrated superior efficacy as the first-line therapy for HER2-positive metastatic breast cancer (MBC), achieving the primary endpoint of progression-free survival. Based on these robust results, the U.S. Food and Drug Administration (FDA) approved this regimen in December 2025. Consistent with these data, the NCCN Guidelines (Version 1.2026) now list pertuzumab plus T-DXd alongside the established taxane-based regimen of pertuzumab, trastuzumab, and a taxane as a first-line treatment option for HER2-positive MBC. This approach has transitioned from an emerging strategy to a standard first-line therapeutic option. Notably, both regimens share a common therapeutic foundation of dual HER2 blockade with trastuzumab and pertuzumab.

Distinct from other recent reviews that focused broadly on antibody–drug conjugate (ADC) resistance, this review specifically concentrates on the molecular mechanisms of resistance to dual HER2 antibody blockade (trastuzumab and/or pertuzumab). The primary value of this article lies in its focused “mapping” between specific biological resistance patterns and subsequent clinical decision-making.

## 2. Trastuzumab

### 2.1. Mechanisms of Action of Trastuzumab

**Figure 1:**
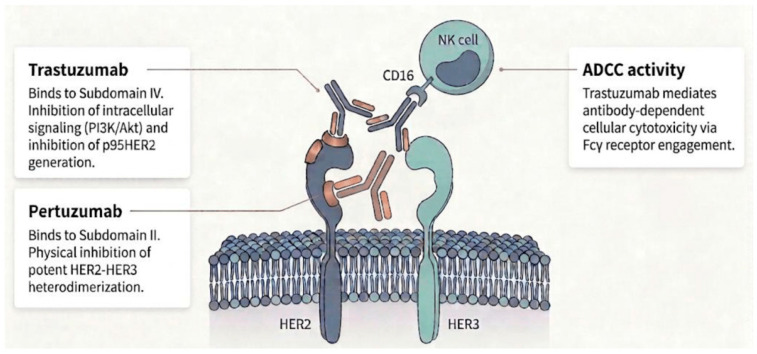
Mechanism of action of trastuzumab and pertuzumab.

Trastuzumab is a humanized IgG1 monoclonal antibody targeting human epidermal growth factor receptor 2 (HER2). It binds specifically to subdomain IV of the extracellular domain and exerts multiple complementary antitumor effects (Figure 1) [9].

First, trastuzumab inhibits receptor-driven intracellular signaling. HER2 forms ligand-independent homodimers or heterodimers with other HER family members, such as HER3, leading to sustained proliferative signaling. The binding of trastuzumab sterically interferes with receptor dimerization and attenuates the downstream activation of the phosphoinositide 3-kinase (PI3K)/Akt pathway, which results in the nuclear accumulation of the cyclin-dependent kinase inhibitor p27^kip1^, induction of G1 cell-cycle arrest, and suppression of tumor-cell proliferation [10].

Second, trastuzumab induces antibody-dependent cellular cytotoxicity (ADCC). The Fc region of trastuzumab engages Fcg receptors (e.g., CD16) expressed on effector cells, including natural killer cells and macrophages. This interaction promotes the recruitment and activation of immune effector cells at the tumor site, leading to the lysis of HER2-overexpressing tumor cells through the release of perforin and granzymes [11].

Third, trastuzumab inhibits the proteolytic cleavage of the HER2 extracellular domain. HER2 is susceptible to shedding by cell-surface metalloproteases such as ADAM10 and ADAM17, generating a truncated constitutively active fragment known as p95HER2, which retains kinase activity and contributes to therapeutic resistance. By binding to subdomain IV, trastuzumab reduces receptor shedding and limits the generation of p95HER2, thereby contributing to sustained antitumor activity [12].

Through these mechanisms—suppression of receptor signaling, activation of immune-mediated cytotoxicity, and inhibition of receptor shedding—trastuzumab exerts multilayered antitumor effects. On this basis, the combination of trastuzumab and chemotherapy demonstrated a significant survival benefit compared with chemotherapy alone [13]. However, in the first-line treatment of metastatic breast cancer, the progression-free survival achieved with trastuzumab plus chemotherapy remained at approximately 12 months [14,15,16], and disease progression due to primary or acquired resistance occurred in many patients.

### 2.2. Mechanisms of Resistance to Trastuzumab

#### 2.2.1. Structural Alterations of the Target Receptor HER2

##### p95HER2

p95HER2, a truncated HER2 fragment lacking the extracellular domain (ECD) that contains the trastuzumab-binding site, is expressed in approximately 30% of HER2-positive breast cancers [17]. p95HER2 is generated either through metalloprotease-mediated shedding of the ECD [18] or through translation from alternative initiation sites within HER2 mRNA [19]. Because p95HER2 lacks subdomain IV, trastuzumab cannot bind or inhibit the receptor. However, the intracellular kinase domain is preserved, allowing for constitutive downstream signaling in a ligand-independent manner [17].

Clinically, p95HER2 expression in metastatic breast cancer has been associated with resistance to trastuzumab and with poorer outcomes [20,21]. In early-stage disease, similar associations have been reported [22]; however, other studies have shown that p95HER2 expression correlated with favorable responses to neoadjuvant regimens that included trastuzumab [23]. The clinical significance of p95HER2 in early breast cancer therefore remains inconsistent and may be influenced by the detection methods, disease stage, treatment context, and underlying tumor biology [7].

##### HER2 Splice Variants (d16-HER2)

Preclinical studies have identified d16-HER2, a splice variant lacking exon 16 of the HER2 transcript, which arises from exon 16 skipping and is expressed in a substantial proportion of HER2-positive breast tumors [7,24]. d16-HER2 forms constitutively active homodimers at the tumor-cell surface and promotes the phosphorylation and activation of SRC kinase (pSRC), thereby enhancing cellular proliferation and tumorigenicity [24,25]. In vitro, d16-HER2 expression has been associated with reduced sensitivity to trastuzumab [26].

In contrast, studies using transgenic mouse models have shown that tumors expressing d16-HER2 may be more sensitive to trastuzumab than those expressing wild-type HER2 [23,25]. This apparent discrepancy may reflect the increased dependence of d16-HER2-driven tumors on SRC-mediated and HER2-dependent signaling, rendering them more vulnerable to HER2 blockade in vivo. Thus, although d16-HER2 may confer resistance in vitro, its strong reliance on HER2 signaling may enhance its susceptibility to trastuzumab in certain in vivo contexts.

##### HER2 Missense Mutations

The overall frequency of HER2 mutations in breast cancer is low; data from The Cancer Genome Atlas indicate a prevalence of approximately 3.5%. The coexistence of HER2 mutations with HER2 gene amplification is uncommon [27], and such mutations are more often detected in metastatic tumors after prior therapy [28]. Representative alterations include *L755S*, *V777L*, and *S310F*.

*L755S* is a recurrent kinase-domain mutation in breast cancer. This alteration enhances the activation of both the MAPK and PI3K–AKT–mTOR pathways and sustains proliferative signaling despite trastuzumab binding [29]. As a result, resistance to both trastuzumab and lapatinib has been reported [30].

*V777L* is another activating mutation within the kinase domain and has been shown in vitro to increase HER2 kinase activity approximately 20-fold compared with the wild type [31]. Clinical observations have also linked this mutation to trastuzumab resistance [32].

*S310F* is located in domain II of the extracellular region. This mutation promotes active heterodimerization with EGFR and may not be adequately suppressed by dimerization blockade with trastuzumab or pertuzumab [33]. Accordingly, *S310F* has been implicated as a potential mechanism of resistance to pertuzumab [33].

##### Reduction in or Loss of HER2 Expression

HER2 status is defined by the ASCO/CAP guidelines (IHC 3+ or ISH positive), and “HER2 loss” refers to a shift to IHC 0, 1+, or 2+/ISH negative after therapy [1], defined as conversion from HER2-positive disease at baseline (IHC 3+ or ISH-positive) to HER2-negative status at reassessment (IHC 0 or 1+, or IHC 2+ with ISH-negative results). The reduction in or loss of HER2 expression after anti-HER2 therapy has been observed in clinical practice. In patients with HER2-positive breast cancer, neoadjuvant trastuzumab combined with anthracycline- and taxane-based chemotherapy achieves a high pathologic complete response rate (51%). However, among patients with residual disease, 32% showed the loss of HER2 gene amplification, which was associated with poorer outcomes [34].

A principal mechanism underlying reduced or absent HER2 expression is thought to be clonal selection driven by intratumoral heterogeneity. Anti-HER2 therapy may preferentially eliminate HER2-dependent clones, allowing for the expansion of preexisting tumor-cell populations with lower HER2 expression or reduced reliance on HER2 signaling.

An additional proposed mechanism is trastuzumab-induced receptor internalization. Sustained antibody binding may promote endocytosis and the intracellular degradation of HER2, leading to decreased receptor density at the cell surface if the degradation exceeds the recycling. However, HER2 is intrinsically resistant to endocytosis; thus, this mechanism alone is unlikely to account for the substantial loss of membrane HER2 expression [35,36].

#### 2.2.2. Steric Hindrance and Epitope Masking by Mucin 4 (MUC4)

Mucin 4 (MUC4), a transmembrane glycoprotein, can form a complex with HER2 and create steric hindrance that limits the physical access of trastuzumab to subdomain IV of the receptor [36]. In studies using the trastuzumab-resistant JIMT-1 cell line, overexpression of MUC4 markedly reduced trastuzumab binding, whereas MUC4 knockdown restored drug sensitivity [37].

Tumor necrosis factor-α (TNF-α) has also been shown to upregulate MUC4 expression, thereby promoting the acquisition of trastuzumab resistance through this mechanism [38].

#### 2.2.3. Constitutive Activation of the PI3K–AKT–mTOR Pathway

##### *PIK3CA* Mutations

*PIK3CA* is an oncogene encoding the p110α catalytic subunit of class IA phosphoinositide 3-kinase (PI3K). Activated mutations, most commonly in the helical and kinase domains, lead to the constitutive production of phosphatidylinositol-3,4,5-trisphosphate (PIP3). This results in the persistent activation of the AKT–mTOR pathway, which promotes cell proliferation, survival, metabolic reprogramming, and resistance to systemic therapies, including endocrine therapy.

*PIK3CA* mutations occur in approximately 20–30% of HER2-positive breast cancers [39] and represent a prototypical mechanism of resistance to HER2-targeted therapy. These mutations result in the constitutive activation of the PI3K–AKT–mTOR signaling axis, thereby uncoupling the downstream pathway activity from the upstream HER2 receptor blockade. Consistent with this biologic mechanism, meta-analyses have demonstrated that tumors harboring *PIK3CA* mutations are associated with significantly lower rates of pathologic complete response (pCR) in the neoadjuvant setting [40] and shorter progression-free survival (PFS) in metastatic disease [41] compared with *PIK3CA*-wild-type tumors. By sustaining downstream signaling despite HER2 inhibition, oncogenic activation of the PI3K pathway diminishes the therapeutic impact of agents such as trastuzumab.

##### Loss and Dysfunction of PTEN

PTEN (phosphatase and tensin homolog) is a key tumor suppressor that negatively regulates the PI3K–AKT–mTOR signaling pathway. As a lipid phosphatase, PTEN dephosphorylates phosphatidylinositol-3,4,5-trisphosphate (PIP3) to phosphatidylinositol-4,5-bisphosphate (PIP2) at the cell membrane, thereby limiting AKT activation and restraining cell proliferation, survival, metabolism, and cell-cycle progression. The loss of PTEN function leads to the accumulation of PIP3 and the constitutive activation of AKT, resulting in persistent mTOR signaling, the suppression of apoptosis, enhanced proliferative signaling, and the acquisition of drug resistance [42]. The loss of PTEN also results in the hyperactivation of the PI3K pathway and has been observed in approximately 59% of trastuzumab-resistant tumors. When considered together, PIK3CA mutations and PTEN loss account for PI3K pathway activation in up to 71% of cases, underscoring its central role as a clinically relevant therapeutic target [43].

##### Downstream Signaling Activation and Effects on Cell-Cycle Regulators

Trastuzumab promotes the nuclear accumulation of p27^kip1^ through the inactivation of AKT, leading to G1 cell-cycle arrest [44,45]. However, when the PI3K pathway is hyperactivated, AKT phosphorylates p27^kip1^ and promotes its degradation, thereby abrogating cell-cycle arrest and sustaining tumor-cell proliferation [46]. Additional alterations in cell-cycle control have also been implicated in trastuzumab resistance. Overexpression of cyclin E [47] and activation of CDK4/6 [48] facilitate G1–S phase transition and may contribute to continued proliferation despite HER2 blockade.

#### 2.2.4. Activation of Bypass Pathways via Alternative Growth Factor Receptors

##### Compensatory Crosstalk Within the HER Family (HER3 and EGFR)

HER3 is a principal dimerization partner of HER2 and EGFR and plays a central role in the activation of the PI3K pathway [49]. Pertuzumab inhibits HER2 dimerization and thereby suppresses this signaling axis [50]. However, the increased expression of neuregulin 1 (NRG1), a ligand for HER3, can restore HER3-mediated signaling and contribute to therapeutic resistance [51,52]. Overexpression of EGFR may also sustain downstream pathway activation through compensatory signaling within the HER receptor family, which has been associated with resistance to trastuzumab [53,54].

##### Alternative Signaling Pathways Mediated by Non-HER Family Receptors

The activation of bypass pathways mediated by receptors outside the HER family is an additional mechanism of trastuzumab resistance.

Enhanced signaling through insulin-like growth factor 1 receptor (IGF-1R) attenuates the antitumor activity of trastuzumab and contributes to resistance [55]. In trastuzumab-resistant cell lines, crosstalk between HER2 and IGF-1R has been observed, allowing persistent HER2 phosphorylation despite the presence of trastuzumab [56].

MET signaling also functions as an alternative pathway. In lapatinib-resistant models, MET activates PI3K–AKT and MAPK–ERK signaling through Src, maintaining downstream signaling after HER2 inhibition [57]. In clinical specimens, increased copy numbers of *MET* or its ligand *HGF* have been associated with the failure of trastuzumab-based therapy [58].

EphA2, a member of the Eph receptor family, signals through ligand-dependent mechanisms and ligand-independent activation via S897 phosphorylation by kinases such as AKT and RSK. It regulates tumor invasion, metastasis, and therapeutic resistance [59,60]. EphA2 upregulation has been linked to acquired trastuzumab resistance, whereas its inhibition restores drug sensitivity in preclinical models; its overexpression in clinical specimens is associated with poor prognosis [61].

AXL, a TAM family receptor activated by Gas6 [62], promotes tumor progression through the induction of epithelial–mesenchymal transition (EMT) and maintenance of stem cell-like properties [63] and is considered a potential therapeutic target in refractory cancers [64]. In HER2-positive breast cancer, AXL overexpression contributes to the resistance to trastuzumab and lapatinib [65], partly through the formation of AXL–HER2 heterodimers that activate PI3K–AKT and MAPK–ERK signaling [66]. AXL expression correlates with EMT features and poor prognosis [66,67].

Notch receptors (Notch1–4) mediate contact-dependent signaling and regulate stem cell maintenance [68]. The HER2 blockade with trastuzumab can induce compensatory Notch activation, supporting the survival and self-renewal of HER2-positive cancer stem cells and potentially contributing to recurrence from residual disease [69,70]. Accordingly, Notch inhibition is being explored as a strategy to overcome resistance.

#### 2.2.5. Impaired Host Immune Response and Changes in the Tumor Microenvironment

Antibody-dependent cellular cytotoxicity (ADCC) mediated through FcγRIIIα (CD16A) on natural killer (NK) cells plays a central role in the antitumor activity of trastuzumab and pertuzumab [11,71]. Clinically, patients carrying the high-affinity FcγRIIIα-158V allele have shown higher response rates and longer progression-free survival (PFS) compared with those carrying the low-affinity 158F allele [72,73]. In addition to innate immunity, adaptive immune responses are also required; types I and II interferon signaling and CD8-positive T-cell-mediated immunity have been shown to be essential components of effective anti-HER2 antibody therapy [74]. Host-related factors that impair immune-mediated cytotoxicity include reduced NK-cell function [75] and the suppression of T-cell activity [76]. Tumor-intrinsic mechanisms of immune escape involve the secretion of transforming growth factor β (TGF-β) [77] and neuromedin U (NmU) [78] and the upregulation of PD-L1 [79]. The tumor microenvironment further contributes to resistance: fibroblast growth factor 5 (FGF5) from tumor-associated fibroblasts can transactivate HER2 via FGFR2, sustaining tumor survival despite therapy [80]. Stromal TGF-β and exosome-mediated signaling likewise promote immune evasion and intracellular reprogramming, thereby attenuating the trastuzumab-mediated antitumor effects [78,81].

#### 2.2.6. Metabolic Adaptation and Enhanced Glycolysis

Trastuzumab-resistant cells exhibit enhanced glycolysis consistent with the Warburg effect [82]. These cells also demonstrate metabolic plasticity, with the ability to shift between glycolysis and oxidative phosphorylation (OXPHOS) to meet energy demands under therapeutic stress [83].

Mechanistically, metabolic reprogramming contributes to resistance. HER2–HSF1-mediated overexpression of lactate dehydrogenase A (LDH-A) enhances glycolytic flux [84] while the upregulation of mitochondrial ATP synthase sustains oxidative phosphorylation; coordination of these pathways supports therapeutic resistance [83]. The silencing of ANKRD44 further augments glycolysis [84]. In parallel, the histone reader ZMYND8 induces c-Myc expression, leading to the upregulation of cytosolic phospholipase A2α (cPLA2α) and the remodeling of glycerophospholipid metabolism, thereby promoting resistance to HER2-targeted antibodies [85]. Collectively, these metabolic adaptations represent potential therapeutic targets for overcoming resistance.

#### 2.2.7. Reinforced Evasion of Apoptosis and Abnormal DNA Damage Response

Acquired resistance to trastuzumab involves the enhanced evasion of apoptosis and disruption of the DNA damage response (DDR). Upregulation of antiapoptotic proteins—including survivin, XIAP, and Bcl-2 family members—often via STAT3 activation, attenuates drug-induced cell death [86,87]. Concurrent alterations in regulators such as ATM and RB1 impair DDR signaling and promote escape from therapy-induced senescence and apoptosis [28,88]. These adaptations sustain tumor cell survival despite HER2 inhibition, ultimately contributing to relapse and resistance.

## 3. Mechanisms of Action of Trastuzumab Plus Pertuzumab

The combination of trastuzumab and pertuzumab significantly prolonged progression-free and overall survival as the first-line therapy for HER2-positive metastatic breast cancer in the CLEOPATRA trial [14], establishing the current standard of care. The clinical efficacy of this regimen is based on complementary molecular mechanisms resulting from binding to distinct epitopes on the HER2 receptor.

Trastuzumab binds to subdomain IV of the HER2 extracellular domain and primarily inhibits ligand-independent homodimerization and proteolytic receptor shedding, thereby suppressing HER2 signaling (Figure 1) [11]. However, its ability to block ligand-dependent HER2–HER3 heterodimerization, one of the most potent drivers of proliferative signaling, is limited.

Pertuzumab binds to subdomain II of the HER2 extracellular domain, known as the “dimerization arm”, which is essential for heterodimer formation with other HER family receptors. By inducing steric hindrance at this interface, pertuzumab directly and effectively inhibits HER2–HER3 heterodimerization that is not adequately suppressed by trastuzumab alone [50,89].

Combined administration therefore suppresses HER2 receptor function from two complementary angles: inhibition of dimerization and stabilization of receptor structure, resulting in a broader blockade of receptor activation. Pertuzumab binding is also thought to be less affected by the steric hindrance caused by MUC4. In the trastuzumab-resistant MUC4-overexpressing JIMT-1 cell line, the binding of pertuzumab has been reported to be preserved [37].

From an immunologic perspective, both trastuzumab and pertuzumab are IgG1 antibodies capable of inducing innate immune-mediated antitumor effects. The simultaneous binding of two antibodies to different epitopes on the same tumor cell increases the density of the Fc regions displayed on the cell surface. This promotes the multivalent engagement of Fcγ receptors (e.g., CD16) on natural killer cells and macrophages, leading to more potent antibody-dependent cellular cytotoxicity compared with either agent alone [90].

### Mechanisms of Resistance to Trastuzumab Plus Pertuzumab

**Figure 2:**
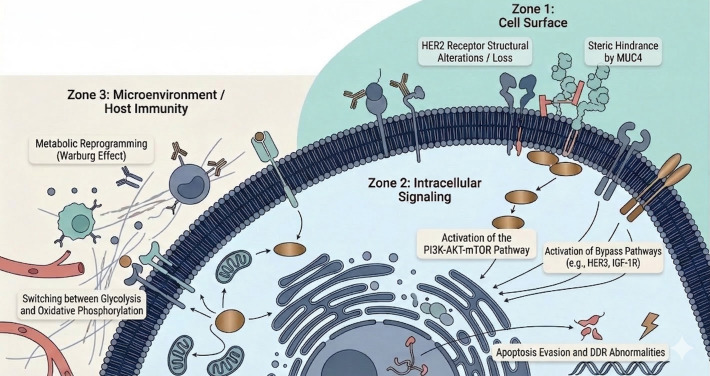
Resistance mechanisms of anti-HER2 antibodies.

Several resistance mechanisms known for trastuzumab remain difficult to overcome, even with the addition of pertuzumab. Both trastuzumab and pertuzumab are monoclonal antibodies targeting HER2, and their mechanisms of resistance frequently overlap (Table 1 and Figure 2). Structural alterations that prevent antibody binding, particularly the expression of p95HER2, remain central mechanisms of resistance. HER2 missense mutations such as L755S, V777L, and S310F can maintain receptor activation and sustain downstream signaling despite dual-antibody therapy. The loss of or marked reduction in HER2 expression represents the effective loss of the therapeutic target and markedly diminishes the activity of anti-HER2 antibodies.

Alterations downstream of the receptor are also critical. Robust activation of the PI3K–AKT pathway can bypass the upstream HER2 blockade. In addition, activation of bypass signaling through receptors and pathways outside the HER family—including IGF-1R, MET, EphA2, AXL, and Notch—can support tumor-cell survival under dual HER2 inhibition.

Resistance mediated by host immunity and the tumor microenvironment is likewise difficult to reverse. FcγRIIIa polymorphisms, impaired NK- or T-cell function, and tumor-derived factors such as TGF-β, neuromedin U, and PD-L1 expression can attenuate antibody-dependent immune mechanisms. Stromal components, including cancer-associated fibroblast-derived FGF5 and TGF-β, as well as exosome-mediated signaling, further contribute to immune evasion and adaptive resistance. Intracellular adaptations—including metabolic reprogramming, epigenetic changes, enhanced evasion of apoptosis, and abnormalities in the DNA damage response—may further limit the treatment efficacy despite pertuzumab coadministration.

Recent preclinical work established resistance models through long-term exposure to trastuzumab plus pertuzumab in four HER2-positive breast cancer cell lines (AU-565, BT-474, EFM-192A, and SK-BR-3). In these models, HER2 expression itself remained largely unchanged, whereas the activation of other HER family receptors, including HER4, and the marked upregulation of MAPK signaling (pERK, pP38) were observed. Proteomic analyses further identified alterations in more than 600 proteins involved in ribosome biogenesis, mitochondrial function, and metabolic reprogramming as potential contributors to resistance development [91].

## 4. Treatment Strategies Based on Clinical Practice Guidelines and the Clinical Role of Trastuzumab Plus Pertuzumab

Current major clinical practice guidelines recommend the combination of trastuzumab, pertuzumab, and a taxane as the standard first-line therapy for HER2-positive metastatic breast cancer [92,93,94], based on the CLEOPATRA trial, which demonstrated significant improvements in progression-free and overall survival. This regimen remains the therapeutic backbone of first-line anti-HER2 treatment. Moreover, as noted above, the combination of pertuzumab and trastuzumab deruxtecan (T-DXd) has been incorporated as a first-line treatment option in the most recent NCCN Clinical Practice Guidelines, where its use is now recommended [93].

The European Society for Medical Oncology (ESMO) Living Guideline for metastatic breast cancer designates T-DXd as the preferred second-line therapy following progression with first-line treatment [92]. T-DXd has shown high response rates and a meaningful survival benefit, establishing its central role in the second-line setting. For patients with active brain metastases not amenable to local therapy, the combination of trastuzumab, tucatinib, and capecitabine is also listed as a recommended regimen alongside T-DXd [92].

In the third-line setting, the treatment selection generally includes whichever of T-DXd or trastuzumab plus tucatinib plus capecitabine has not yet been used. Trastuzumab emtansine (T-DM1) is positioned as a subsequent option. Beyond these lines, the guidelines from the European Society for Medical Oncology, the National Comprehensive Cancer Network, and the American Society of Clinical Oncology recommend continued sequential use of available HER2-directed therapies [92,93,94].

This sequential treatment strategy is designed to maximize tumor control by employing agents with distinct mechanisms of action—monoclonal antibodies, tyrosine kinase inhibitors, and antibody–drug conjugates—at different stages of treatment, thereby addressing heterogeneous and evolving resistance mechanisms. Trastuzumab plus pertuzumab serves as the foundation of this approach by providing comprehensive HER2 blockade and immune-mediated antitumor activity, establishing initial disease control that enables subsequent lines of therapy.

### 4.1. Second- and Third-Line Therapies for HER2-Positive Metastatic Breast Cancer

#### 4.1.1. Trastuzumab Deruxtecan (T-DXd)

Trastuzumab deruxtecan (T-DXd) is an HER2-directed next-generation antibody–drug conjugate (ADC) with a bystander effect. It has a drug-to-antibody ratio (DAR) of eight and consists of trastuzumab linked via a cleavable tetrapeptide linker to a topoisomerase I inhibitor payload (an exatecan derivative) [95]. Among the previously described mechanisms of resistance to trastuzumab and pertuzumab, preservation of sufficient HER2 expression to allow for the binding and internalization of T-DXd is the key determinant of activity. Constitutive activation of the PI3K–AKT–mTOR pathway (including PIK3CA mutations, PTEN loss, and downstream pathway activation), activation of bypass signaling through alternative receptors (EGFR/HER3, IGF-1R, MET, AXL, EphA2, and Notch), HER2 kinase-domain mutations (e.g., L755S, V777L), and immune-dependent mechanisms of trastuzumab resistance (such as Fcg receptor polymorphisms or impaired NK- and T-cell function) may still be susceptible to the cytotoxic payload of T-DXd. In addition, the bystander effect enables antitumor activity in tumors with intratumoral HER2 heterogeneity, representing a major therapeutic advantage.

In contrast, the activity is likely to be limited when HER2 expression is completely lost, when extracellular domain alterations impair the binding or internalization of T-DXd [96], when marked MUC4 overexpression results in extensive epitope masking, or when tumors are predominantly driven by p95HER2 with minimal residual full-length HER2-expressing clones. The sensitivity may also be reduced in the presence of profound intracellular adaptations including metabolic reprogramming, enhanced antiapoptotic signaling, and abnormalities in the DNA damage response (DDR) [97].

Clinical efficacy was demonstrated in the DESTINY-Breast03 trial [98], a phase 3 study comparing T-DXd with T-DM1 in patients with HER2-positive metastatic breast cancer previously treated with trastuzumab and a taxane. T-DXd significantly prolonged the progression-free survival (median, 29 months vs. 7.2 months; hazard ratio [HR], 0.30; 95% CI, 0.24–0.38; *p* < 0.000001) and improved overall survival (median, 52.6 months vs. 42.7 months; HR, 0.73; 95% CI, 0.56–0.94). The objective response rate was 79.7% with T-DXd and 34.2% with T-DM1. Interstitial lung disease occurred in 16.7% of patients receiving T-DXd.

Approximately 60% of patients in this study had previously received pertuzumab, and about half were treated in the second-line setting. In the T-DM1 group, 32.3% of patients subsequently received T-DXd. On the basis of these results, T-DXd has become the standard second-line therapy for HER2-positive metastatic breast cancer in major clinical practice guidelines.

The efficacy of trastuzumab deruxtecan in patients receiving third-line or later therapy was also demonstrated in the DESTINY-Breast02 trial [99].

#### 4.1.2. Tucatinib Plus Trastuzumab and Capecitabine

Tucatinib is an ATP-competitive tyrosine kinase inhibitor with high selectivity for HER2. It suppresses the phosphorylation of HER2 and HER3 and inhibits downstream PI3K–AKT and MAPK signaling. Its selectivity for HER2 is approximately 1000-fold greater than for EGFR, allowing for effective HER2 pathway inhibition with reduced EGFR-related toxic effects such as rash and diarrhea [100]. In preclinical models, tucatinib has shown activity against truncated HER2 forms, including p95HER2, which are implicated in trastuzumab resistance, and has demonstrated higher central nervous system (CNS) penetration and intracranial antitumor activity than lapatinib or neratinib [101].

Among the known mechanisms of resistance to trastuzumab and pertuzumab, those potentially addressed by tucatinib include structural alterations such as p95HER2, steric hindrance caused by MUC4, extracellular domain mutations (e.g., *S310F*) that impair antibody binding, and reduced immune-mediated activity such as diminished ADCC. In contrast, strong downstream activation of the PI3K–AKT pathway, bypass signaling through receptors such as IGF-1R, loss of or marked reduction in HER2 expression, metabolic adaptation, and enhanced antiapoptotic signaling or DNA damage response abnormalities are less likely to be overcome by tucatinib alone.

Clinical benefits were established in the HER2CLIMB trial [102]. This phase 3 study enrolled patients with HER2-positive metastatic breast cancer previously treated with trastuzumab, pertuzumab, and T-DM1 and notably included patients with active brain metastases. Patients were randomly assigned in a 2:1 ratio to receive tucatinib plus trastuzumab plus capecitabine or placebo plus trastuzumab plus capecitabine. The tucatinib regimen significantly improved the progression-free survival (median, 7.8 vs. 5.6 months; HR, 0.54; 95% CI, 0.42–0.71; *p* = 0.0163) and the OS (median, 24.7 vs. 19.2 months; HR, 0.73; 95% CI, 0.59–0.90; *p* = 0.004). In the subgroup with brain metastases, tucatinib significantly prolonged the CNS-PFS (median, 9.9 vs. 4.2 months; HR, 0.32; 95% CI, 0.22–0.48; *p* < 0.00001) and OS (median, 18.1 vs. 12.0 months; HR, 0.58; 95% CI, 0.40–0.85; *p* = 0.005) [103]. This regimen was the first HER2-targeted therapy to demonstrate an overall survival benefit in a prospective trial that included patients with active brain metastases, and it has substantially influenced the treatment algorithm for HER2-positive metastatic breast cancer. Diarrhea and grade 3 or higher elevations in aminotransferase levels were more common in the tucatinib group than in the placebo group.

#### 4.1.3. Trastuzumab Emtansine (T-DM1)

Trastuzumab emtansine (T-DM1) is a first-generation antibody–drug conjugate (ADC) consisting of the humanized anti-HER2 monoclonal antibody trastuzumab linked to the microtubule inhibitor DM1, a maytansine derivative, via a stable noncleavable linker (SMCC). The drug-to-antibody ratio (DAR) is approximately 3.5, and T-DM1 does not exert a bystander effect [104].

Although T-DM1 shares certain features with T-DXd as an ADC, important differences influence its ability to overcome resistance. Its activity is limited in tumors with low HER2 expression or marked intratumoral heterogeneity, and its efficacy is reduced in tumors with structural alterations such as p95HER2 [105]. Strong activation of the PI3K–AKT pathway has also been associated with the diminished activity of T-DM1 [106], whereas the impact of similar alterations appears less pronounced with T-DXd [107]. In addition, the antitumor activity of T-DM1 relies in part on the antibody-dependent cellular cytotoxicity, suggesting that impaired host immune function may further reduce its efficacy compared with T-DXd [108].

Clinical outcomes after prior trastuzumab and pertuzumab exposure can be inferred from the control arm of the DESTINY-Breast03 trial [98], which evaluated T-DXd versus T-DM1. In that study, the median progression-free survival with T-DM1 was 7.2 months, the median overall survival was 42.7 months, and the objective response rate was 34.2%. Although cross-trial comparisons should be interpreted cautiously, these outcomes appear lower than those reported in the EMILIA trial, in which T-DM1 achieved a median progression-free survival of 9.6 months and a response rate of 43.6% [109]. Approximately 60% of patients in DESTINY-Breast03 had previously received pertuzumab, reflecting a population with more advanced resistance to HER2-directed therapy. Adverse events that commonly occur with T-DM1 include thrombocytopenia and hepatotoxicity.

### 4.2. Treatment Options Beyond the Third Line

#### 4.2.1. Neratinib Plus Capecitabine

Neratinib is an irreversible pan-HER tyrosine kinase inhibitor targeting EGFR (HER1), HER2, and HER4. Compared with the reversible inhibitor lapatinib, neratinib provides more potent and sustained inhibition of HER family signaling [110].

Among the mechanisms of resistance to trastuzumab and pertuzumab, the activity of neratinib overlaps with that of other HER-directed TKIs but has some distinctions. In particular, compensatory signaling within the HER family—such as activation mediated through EGFR or HER4—may be more effectively suppressed by neratinib than by HER2-selective inhibitors such as tucatinib due to its broader pan-HER activity.

Its clinical efficacy was evaluated in the phase 3 NALA trial [111], which enrolled patients with previously treated HER2-positive metastatic breast cancer. Neratinib plus capecitabine was compared with lapatinib plus capecitabine; approximately 40% of patients had prior exposure to trastuzumab plus pertuzumab. Neratinib significantly improved the PFS compared with lapatinib (HR, 0.76; 95% CI, 0.63–0.93; stratified log-rank *p* = 0.0059), and no significant overall survival benefit was observed (HR, 0.88; 95% CI, 0.72–1.07; *p* = 0.2098). The cumulative incidence of intervention for central nervous system metastases was lower in the neratinib group (22.8% vs. 29.2%; *p* = 0.043). The objective response rate did not differ significantly between groups (32.8% vs. 26.7%; *p* = 0.1201). Diarrhea (83% vs. 66%) and nausea (53% vs. 42%) were more frequent with neratinib, but the rates of treatment discontinuation and health-related quality of life were similar between groups.

#### 4.2.2. Margetuximab Plus Chemotherapy (Capecitabine, Eribulin, Gemcitabine, or Vinorelbine)

Margetuximab is an Fc-engineered chimeric IgG1κ anti-HER2 monoclonal antibody designed to enhance antibody-dependent cellular cytotoxicity (ADCC). It targets subdomain IV of the HER2 extracellular domain, overlapping with the trastuzumab epitope. The Fc region contains amino acid substitutions that increase affinity for the activating Fcγ receptor FcγRIIIa (CD16A) and decrease affinity for the inhibitory FcγRIIb (CD32B), resulting in enhanced NK cell-mediated ADCC [112].

Its clinical efficacy was evaluated in the phase 3 SOPHIA trial [113], which enrolled patients with HER2-positive metastatic breast cancer previously treated with at least two lines of therapy. Patients were randomly assigned in a 1:1 ratio to receive margetuximab plus the physician’s choice of chemotherapy or trastuzumab plus the physician’s choice of chemotherapy. All patients had prior exposure to trastuzumab and pertuzumab. Margetuximab significantly improved the PFS compared with trastuzumab (hazard ratio, 0.71; 95% CI, 0.58–0.86; *p* < 0.001; median, 5.7 vs. 4.4 months) and increased the objective response rate (25% vs. 14%; *p* < 0.001). No significant improvement in OS was observed (HR, 0.95; 95% CI, 0.77–1.17; *p* = 0.620; median, 21.6 vs. 21.9 months) [114].

In a prespecified exploratory analysis of the CD16A-158 polymorphism (V, high-affinity; F, low-affinity), patients with the CD16A-158FF genotype showed a trend toward improved OS with margetuximab compared with trastuzumab (median, 23.6 vs. 19.2 months; HR, 0.72; 95% CI, 0.52–1.00). In contrast, among patients with the CD16A-158VV genotype, the OS appeared to be longer with trastuzumab than with margetuximab (median, 31.1 vs. 22.0 months; HR, 1.77; 95% CI, 1.01–3.12) [114]. These findings suggest the potential for the genotype-informed selection of anti-HER2 antibodies based on Fcγ receptor polymorphisms.

#### 4.2.3. Retreatment with Pertuzumab Plus Trastuzumab

In principle, retreatment with the same combination of anti-HER2 antibodies might be expected to have limited efficacy once resistance to trastuzumab and pertuzumab has developed. In clinical practice, however, anti-HER2 antibodies are administered with different chemotherapy partners across lines of therapy, and intervening treatments with distinct mechanisms, such as T-DM1, may alter the tumor biology. These factors raise the possibility that the dual HER2 blockade may regain activity in later lines.

Preclinical studies have shown that in T-DM1–resistant cell lines, the presence of neuregulin increases the phosphorylation of HER2 and AKT. In these models, the combination of pertuzumab and trastuzumab suppressed cell proliferation and xenograft tumor growth more effectively than trastuzumab alone [115].

Their clinical efficacy was evaluated in the phase 3 PRECIOUS trial [116], which assessed retreatment with pertuzumab plus trastuzumab in patients with HER2-positive metastatic breast cancer previously treated with this combination in the first- or second-line setting. Pertuzumab was not included in the regimen immediately preceding retreatment, and 97% of patients had received prior T-DM1. Patients were randomly assigned to pertuzumab plus trastuzumab plus the physician’s choice of chemotherapy or trastuzumab plus the physician’s choice of chemotherapy. Investigator-assessed PFS, the primary end point, was significantly prolonged with pertuzumab retreatment (median, 5.3 vs. 4.2 months; HR, 0.76; upper limit of the 95%CI, 0.967; *p* = 0.022). The objective response rates were similar (19.5% vs. 20.7%), but an improvement in the OS was suggested (median, 36.2 vs. 26.5 months; HR, 0.73; upper limit of the 95% CI, 0.97) [117]. No deterioration in the health-related quality of life was observed with the addition of pertuzumab.

Findings from trials such as CLEOPATRA [14], PHEREXA [118], EGF104900 [117], and HER2CLIMB [102] suggest that dual HER2 blockade may confer a higher relative benefit in OS than in progression-free survival compared with single-agent HER2 inhibition. Accordingly, dual HER2 blockade in later treatment lines may be considered as one of the therapeutic options.

#### 4.2.4. Other Regimens

Additional treatment options include trastuzumab plus chemotherapy, lapatinib plus capecitabine, and lapatinib plus trastuzumab. Prospective data for trastuzumab plus chemotherapy after prior trastuzumab and pertuzumab exposure are available from the control arms of trials such as HER2CLIMB [102], SOPHIA [113], and PRECIOUS [116]. In these studies, the median progression-free survival ranged from 4.2 to 5.6 months, the objective response rates from 16% to 23%, and the median overall survival from 17.4 to 26.5 months.

Prospective results for lapatinib plus capecitabine were reported in the NALA trial [112], with a median progression-free survival of 6.6 months, an objective response rate of 26.7%, and a median overall survival of 18.7 months. In contrast, prospective trial data specifically evaluating lapatinib plus trastuzumab after prior pertuzumab plus trastuzumab have not been clearly established.

**Figure 3:**
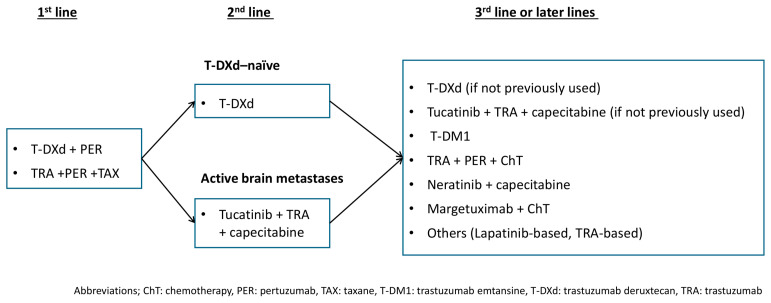
Treatment algorithm for HER2-positive metastatic breast cancer according to current clinical evidence.

Figure 3 illustrates the treatment sequence for HER2-positive metastatic breast cancer based on recent clinical evidence. In addition, representative phase 3 trials conducted after disease progression on trastuzumab plus pertuzumab are summarized in Table 2.

## 5. Clinical Applicability After Pertuzumab–Trastuzumab Therapy: Biomarkers and Treatment Decision-Making

Beyond guideline-based recommendations, therapeutic decisions after trastuzumab–pertuzumab therapy should be informed by the evolving clinical context and biomarker profile. Here, we outline a framework for such biomarker-guided decision-making. Table 3 summarizes the recommended treatment strategies after trastuzumab plus pertuzumab therapy, based on the emerging clinical phenotypes and biomarker alterations, together with their underlying rationale.

To capture the biomarker evolution and emerging mechanisms of resistance after trastuzumab–pertuzumab therapy, a repeat biopsy is essential. The principal time point at which a re-biopsy should be considered is at disease progression during or after first-line trastuzumab plus pertuzumab therapy [120,121].

When metastatic lesions are accessible, tissue biopsy should be performed to reassess HER2 expression and evaluate intratumoral heterogeneity. If HER2 expression is retained—even in the presence of heterogeneity—agents such as trastuzumab deruxtecan (T-DXd) remain compelling therapeutic options [122,123]. However, the interpretation of HER2 loss on tissue biopsy requires caution. A single-site biopsy may be subject to sampling bias arising from spatial intratumoral heterogeneity, potentially resulting in an apparent loss of HER2 expression rather than true biologic conversion [121,124,125].

When tissue sampling is not feasible, or when a more comprehensive assessment of resistance mechanisms is warranted, circulating tumor DNA (ctDNA) analysis may be used to identify alterations such as PIK3CA mutations or HER2 kinase domain mutations. In cases of discordance between the primary tumor and metastatic sites, treatment decisions should generally be guided by the most recent metastatic biopsy [120,126,127].

If HER2 expression is lost, therapeutic strategies beyond HER2-targeted therapy should be considered [128]. Conversely, the detection of HER2 mutations or PIK3CA mutations may support a transition to mutation-directed therapies, including tyrosine kinase inhibitors or antibody–drug conjugates. Thus, a strategy predicated on dynamic target reassessment—reflecting the temporal evolution of tumor biology—represents a cornerstone of personalized sequential therapy.

Ideally, the selection of subsequent therapy should be guided by the underlying mechanisms of resistance. In particular, the detection of driver mutations or dominant resistance pathways through liquid biopsy may facilitate the implementation of molecularly targeted therapies, representing a promising strategy for precision treatment. However, the mechanisms underlying acquired resistance are complex and rarely attributable to a single pathway; instead, multiple mechanisms often interact to drive clinical resistance. Consequently, the selection of subsequent therapies in clinical practice is primarily guided by the available clinical evidence.

## 6. Novel Anti-HER2 Agents in Development

Multiple novel agents are in development to overcome the resistance to established anti-HER2 therapies [129,130].

Bispecific antibodies (BsAbs) are designed to target two antigens simultaneously in order to enhance antitumor activity. Constructs targeting HER2/HER2, HER2/HER3, HER2/CD3, and HER2/CD16 are under investigation. These agents are engineered to augment immune-mediated mechanisms such as ADCC, antibody-dependent cellular phagocytosis, and complement-dependent cytotoxicity, as well as to enhance the blockade of oncogenic signaling. Examples reported to date include MBS301, zanidatamab, KN026, zenocutuzumab, p95HER2 × CD3 bispecific antibodies, and HER2 × CD16 bispecific antibodies.

Several next-generation tyrosine kinase inhibitors are also being developed. These include irreversible pan-HER inhibitors such as pyrotinib and poziotinib, reversible pan-HER inhibitors such as epertinib (S-222611), and highly selective HER2 inhibitors such as DZD1516, which is being explored for improved central nervous system penetration.

Several novel ADCs are in clinical development. Examples include trastuzumab duocarmazine (SYD985), which uses an alkylating payload; ARX788, which employs an amberstatin-based tubulin inhibitor; and disitamab vedotin (RC48), which delivers the microtubule inhibitor MMAE. These emerging agents represent next-generation HER2-targeted therapies designed to address diverse resistance mechanisms encountered with current treatments.

## 7. Conclusions

The treatment landscape for HER2-positive metastatic breast cancer has improved substantially with the introduction of trastuzumab and pertuzumab; however, acquired resistance remains a major clinical challenge. Resistance mechanisms are multilayered and include structural alterations in or loss of HER2 expression, constitutive activation of the PI3K–AKT–mTOR pathway, signaling bypass through alternative receptors, and complex contributions from the tumor microenvironment and metabolic reprogramming.

To address these diverse mechanisms, sequential therapy using agents with distinct modes of action is essential. Trastuzumab deruxtecan (T-DXd), now central in the second-line setting, demonstrates strong cytotoxic activity and a bystander effect, enabling efficacy in tumors with intratumoral heterogeneity or downstream pathway activation. The selective HER2 tyrosine kinase inhibitor tucatinib provides an additional strategy, particularly for disease involving the central nervous system, which may overcome specific mechanisms that limit antibody-based therapies. Trastuzumab emtansine (T-DM1) remains an effective option, although reduced activity may be observed in tumors with low HER2 expression or PI3K pathway alterations.

In later lines, additional HER2-directed agents—including pan-HER inhibitors and Fc-engineered antibodies with enhanced ADCC—extend the opportunity to maintain HER2-targeted treatment. Retreatment with pertuzumab under selected conditions may also offer clinical benefit.

Overall, durable disease control in HER2-positive metastatic breast cancer depends on optimizing sequential anti-HER2 therapy according to the evolving resistance mechanisms. The ongoing development of novel antibody–drug conjugates and bispecific antibodies is expected to further expand strategies capable of addressing tumor heterogeneity and immune evasion.

## Figures and Tables

**Table 1:** Strength of evidence for trastuzumab ± pertuzumab resistance mechanisms.

Category	Mechanism of Resistance to Trastuzumab ± Pertuzumab	Clinical Significance
Consistent clinical evidence	*PIK3CA* mutations	Lower pCR rate in neoadjuvant setting and shorter PFS in clinical trials and meta-analysis [39,40,41,42,43]
	*PTEN* loss	
	HER2 kinase domain mutations	Identified in clinical samples and correlated with clinical resistance to trastuzumab [29,30,31,32]
	Loss of HER2 expression	Identified in clinical samples and correlated with poor outcome [34]
	EGFR overexpression	Identified in clinical samples and correlated with clinical resistance to trastuzumab [53,54]
	Low-affinity FcγRIIIα-158F allele	Identified in clinical samples and correlated with clinical resistance to trastuzumab [72,73]
Context-dependent/Inconsistent	p95HER2	Resistance factor in MBC; inconsistent findings in EBC [17,18,19,20,21,22,23]
	*MET*/*HGF* amplification	Clinical association suggested; large-scale validation needed [57,58]
	EphA2	Clinical association suggested; large-scale validation needed [59,60,61]
	AXL overexpression	Clinical association suggested; large-scale validation needed [65,66,67]
Predominantly preclinical	d16-HER2	Inconsistent between mouse and in vitro models; clinical relevance unclear [25,26]
	HER2 extracellular domain mutations	Cell line-based evidence; clinical validation needed [33]
	MUC4	Cell line-based evidence; clinical validation needed [36,37,38]
	Cyclin E overexpression/CDK4/6 activation	Cell line-based evidence; clinical validation needed [47,48]
	Neureglin 1	Cell line-based evidence; clinical validation needed [51,52]
	FGF5, TGF-β, exosome	Cell line-based evidence; clinical validation needed [78,80,81]
	Metabolic reprogramming	Cell line-based evidence; clinical validation needed [82,83,84,85]
	DNA damage response (*ATM*, *RB1*)	Preclinical novel mechanism (acquired resistance) [88]
	Notch signaling	Hypothetical CSC maintenance mechanism [68,69,70]

Abbreviations; CDK; cyclin-dependent kinase, CSC: cancer stem cell, EBC: early breast cancer, FGF: fibroblast growth factor, TGF: tumor forming growth factor, HGF: hepatocyte growth factor; MBC: metastatic breast cancer, pCR: pathological complete response, PFS: progression-free survival.

**Table 2:** Recent phase III trials of subsequent anti-HER2 therapies after progression on trastuzumab plus pertuzumab in HER2-positive metastatic breast cancer.

Trials	Prior Anti-HER2 Therapy	Experimental Arm (n) Control Arm (n)	PFS, Median	ORR	OS, Median	Characteristic AEs in Experimental Arm
DESTINY-Breast03 [98]	TRA 99.6% PER 61.1%	T-DXd (261) T-DM1 (263)	28.8 M 6.8 M HR 0.33 *p* < 0.000001	78.5% 35.0% Nominal *p* < 0.0001	NR NR HR 0.64 *p* < 0.0037	ILD Nausea, vomiting
DESTINY-Breast02 [99]	TRA 99.8% PER 80.0% T-DM1 99.8%	T-DXd (406) TPC (202)	17.8 M 6.9 M HR 0.36 *p* < 0.0001	69.7% 29.2% *p* < 0.0001	39.2 M 26.5 M HR 0.66 *p =* 0.0021	ILD Nausea, vomiting
HER2CLIM [102,103]	TRA 100% PER 99.8% T-DM1 100%	Tucatinib + TRA + Cape (410) Plac. + TRA + Cape (202)	7.8 M 5.6 M HR 0.66 *p* < 0.001	41% 23% *p* = 0.00008	21.9 M 17.4 M *p* = 0.005	Diarrhea, nausea stomatitis PPE syndrome
SOPHIA [113,114]	TRA 100% PER 91.2% T-DM1 100%	Margetuximab + ChT (266) TRA + ChT (270)	5.7 M 4.4 M HR 0.71 *p* < 0.001	25.2% 13.7% *p* = 0.0006	21.6 M 19.8 M HR 0.89 *p* = 0.33	Infusion-related reaction Fatigue
NALA [111]	TRA 100% PER 42% T-DM1 54%	Neratinib + Cape (307) Lapatinib + Cape (314)	8.8 M 6.6 M HR 0.76 *p* = 0.0059	32.8% 26.7% *p* = 0.12	24.0 M 22.2 M HR 0.88	Diarrhea Nausea, vomiting
PRECIOUS [116,119]	TRA 99.1% PER 99.1% T-DM1 97.7%	PER + TRA + ChT (108) TRA + ChT (109)	5.3 M 4.2 M HR 0.76 *p* = 0.022	19.5% 20.7% OR 0.96	36.2 M 26.5 M HR 0.73	Diarrhea

Abbreviations; AE: adverse event, Cape: capecitabine, ChT: chemotherapy, HR: hazard ratio, ILD: interstitial lung disease, n: number, NR: not reached, ORR: objective response rate, OS: overall survival, PER: pertuzumab, PFS: progression-free survival, Plac: placebo, T-DM1: trastuzumab emtansine, T-DXd: trastuzumab deruxtecan, TPC: treatment of physician’s choice, TRA: trastuzumab.

**Table 3:** Clinical state/molecular biomarkers and corresponding therapeutic strategies.

Clinical State/Molecular Biomarkers	Inferred Resistance Mechanism	Recommended Strategies	Rationale
CNS metastases	Blood–brain barrier permeability, expansion of resistant clone	Tucatinib + Trastuzumab + Capecitabine	Significant improvements in OS and PFS have been demonstrated in patients with brain metastases [102,103]
		T-DXd	Clinically meaningful and improvements in OS and PFS have been observed in patients with brain metastases [98]
Intratumoral HER2 heterogeneity	Clonal selection	T-DXd	Bystander effect [95]
Loss or attenuation of HER2 expression	Target loss	T-DXd	Significant improvements in OS and PFS have been demonstrated in patients with HER2-low/HER2-ultralow disease [96]
*PIK3CA* mutations/*PTEN* loss	Constitutive activation of downstream signaling pathways	T-DXd	Overcomes downstream signaling dependence via potent cytotoxicity [107]
HER2 kinase domain mutations: L755S, V777L	Constitutive receptor activation	T-DXd	T-DXd payload-mediated cytotoxicity [31]
		Neratinib	Irreversible TKI inhibition [31,32,33,34,35,36,37,38,39,40,41,42,43,44,45,46,47,48,49,50,51,52,53,54,55,56,57,58,59,60,61,62,63,64,65,66,67,68,69,70,71,72,73,74,75,76,77,78,79,80,81,82,83,84,85,86,87,88,89,90,91,92,93,94,95,96,97,98,99,100,101,102,103,104,105,106,107,108,109,110]
p95HER2 expression	HER2 ECD deletion	Tucatinib, neratinib	Unaffected by ECD loss due to direct kinase inhibition [101]
FcγRIIIα-158F polymorphism	Impaired ADCC via low-affinity FcγR	Margetuximab	Fc-engineered antibodies enhance ADCC despite low-affinity FcγR [112,113,114]

Abbreviations: ADCC: antibody-dependent cellular cytotoxicity, CNS: central nervous system, ECD: extracellular domain, OS: overall survival, PFS: progression-free survival, T-DXd: trastuzumab deruxtecan, TKI: tyrosine kinase inhibitor.

## Data Availability

No new data were created or analyzed in this study. Data sharing is not applicable to this article.
